# Arsenate reduction coupled to anaerobic oxidation of methane by members of the Methanoperedenaceae

**DOI:** 10.1093/ismejo/wrag251

**Published:** 2026-09-26

**Authors:** Georgina H Joyce, Xueqin Zhang, Andy O Leu, Yosephine Gumulya, Jianhua Guo, Gene W Tyson, Shihu Hu, Simon J McIlroy

**Affiliations:** Centre for Microbiome Research, School of Biomedical Sciences, Queensland University of Technology, Translational Research Institute, Woolloongabba 4102, Australia; Australian Centre for Water and Environmental Biotechnology, The University of Queensland, St Lucia 4072, Australia; Centre for Microbiome Research, School of Biomedical Sciences, Queensland University of Technology, Translational Research Institute, Woolloongabba 4102, Australia; Centre for Microbiome Research, School of Biomedical Sciences, Queensland University of Technology, Translational Research Institute, Woolloongabba 4102, Australia; Australian Centre for Water and Environmental Biotechnology, The University of Queensland, St Lucia 4072, Australia; Centre for Microbiome Research, School of Biomedical Sciences, Queensland University of Technology, Translational Research Institute, Woolloongabba 4102, Australia; Australian Centre for Water and Environmental Biotechnology, The University of Queensland, St Lucia 4072, Australia; Centre for Microbiome Research, School of Biomedical Sciences, Queensland University of Technology, Translational Research Institute, Woolloongabba 4102, Australia

**Keywords:** anaerobic oxidation of methane, anaerobic methanotrophic archaea, arsenic, biogeochemical cycles

## Abstract

Anaerobic methanotrophic ‘*Candidatus* Methanoperedenaceae’ play a key role in mitigating methane emissions from freshwater sediments. Members of the family are metabolically versatile, with species coupling anaerobic oxidation of methane (AOM) to the reduction of nitrate and metal oxides. Metagenomic analyses have indicated further uncharacterized metabolic diversity, including the potential for AOM coupled to arsenate reduction (As-AOM). While As-AOM has been confirmed as an important methane sink and contributor to arsenic mobilization in freshwater environments, the underlying microbiological mechanisms remain to be empirically demonstrated. In this study stoichiometric coupling of dissimilatory arsenate reduction to methane oxidation was demonstrated using enrichment cultures of the uncultured type species ‘*Ca*. Methanoperedens nitroreducens’. Meta-omics revealed that arsenate oxidoreductases were exclusively encoded by ‘*Ca.* M. nitroreducens’ and upregulated under As-AOM conditions, confirming this species was directly responsible. These findings expand our understanding of ‘*Ca.* Methanoperedenaceae’ physiology and demonstrate microbial mechanisms underpinning arsenic mobilization in freshwater systems, with important implications for climate and public health.

## Introduction

‘*Candidatus* Methanoperedenaceae’ are globally distributed anaerobic methanotrophic archaea (ANME) that play a critical role in mitigating the atmospheric release of methane in freshwater environments. Unlike marine-associated ANME-lineages which typically depend on syntrophic partnerships with sulfate-reducing bacteria, ‘*Ca.* Methanoperedenaceae’ are metabolically versatile, having been demonstrated to independently couple anaerobic oxidation of methane (AOM) to the reduction of nitrate and metal oxides [1–5]. Comparative genomic analysis has identified that some members of the family may utilize additional terminal electron acceptors for AOM, including selenate, elemental sulphur and arsenate [6].

Arsenate reduction coupled to AOM (As-AOM) is estimated to account for up to ~50% of the mobilization of arsenic from wetland soils, making it an important microbial process contributing to freshwater arsenic contamination [7–9]. The detection of ‘*Ca.* Methanoperedenaceae’ in soils where As-AOM occurs has led to their suggested involvement in mediating this biogeological process [9]. Several ‘*Ca.* Methanoperedenaceae’ metagenome assembled genomes (MAGs), including that of the type species ‘*Ca.* Methanoperedens nitroreducens’, have been reported to encode putative oxidoreductases most homologous to the bacterial arsenate reductase (*arr*) and anaerobic arsenite oxidoreductase (*arx*). Arr and Arx are members of a diverse superfamily of iron–sulphur molybdoenzymes that mediate respiratory roles in prokaryotes. Similar to the bacterial nitrate reductase complex (narGH) encoded by nitrate-reducing type-species ‘*Ca*. M. nitroreducens’, Arr and Arx typically consist of a heterodimeric a_1_b_1_ complex that is membrane-bound and exported to pseudo/periplasm *via* the twin-arginine secretory system [10]. Both Arr and Arx harbour similar gene-cluster arrangement and can operate bidirectionally *in vitro* under thermodynamically appropriate conditions [11]. The role of these putative oxidoreductases for ‘*Ca.* Methanoperedenaceae’ is unclear, particularly as they form separate phylogenetic clades to functionally characterized Arr/Arx genes, which could indicate functional divergence [6, 10]. Moreover, it was found that ‘*Ca*. M. nitroreducens’ ArxA harbours an active site motif (SGRN(R/Q)G) different to canonical ArxA (XGRGWG) [10]. It is possible that ‘*Ca.* Methanoperedenaceae’ use Arr, Arx, or both, to transfer methane-derived electrons to arsenate.

A previous study found that transcription of ‘*Ca.* Methanoperedenaceae’ methyl-coenzyme M reductase (*mcrA*), arsenate reductase (*arrA*) were correlated with methane oxidation rates in wetland soils, though the *arrA* transcripts were deemed bacterial and unlinked to the ANME species present, leading the authors to suggest an undefined syntrophic partnership between the ANME and bacterial arsenate reducers [9]. A separate study found a correlation between the expression of total community *arrA* transcripts (including those linked to ‘*Ca*. Methanoperedenaceae’) and ‘*Ca.* Methanoperedenaceae’-like *mcrA*, suggesting direct involvement of these archaea in As-AOM in rice paddy sediments [12]. However, *arrA* gene expression was dominated (~95%) by other organisms, making it unclear whether As-AOM by ‘*Ca.* Methanoperedenaceae’ contributed to the biogeochemical transformations observed. Collectively, these studies support the hypothesis that members of ‘*Ca.* Methanoperedenaceae’ play a role in As-AOM in the environment; however, direct evidence and a clear understanding of the underlying microbial mechanisms are still lacking.

In this study, comparative genomic analysis of publicly available ‘*Ca.* Methanoperedenaceae’ MAGs was applied to assess the prevalence of arsenate metabolism potential in the family. Batch incubation studies were then performed with an enrichment of the nitrate reducing type species ‘*Ca.* Methanoperedens nitroreducens’, which encodes both a putative arsenate reductase (*arr*) and an anaerobic arsenite oxidoreductase (*arx*) complex. Nitrate was replaced with arsenate as the sole electron acceptor for AOM, and the ability of the enrichment to perform As-AOM was assessed using biochemical, metagenomic, and metatranscriptomic analyses. This work delivers experimental demonstration that a microorganism can directly mediate As-AOM and examines the adaptive strategies supporting this metabolism for ‘*Ca.* Methanoperedenaceae’.

## Materials and methods

### ‘*Ca.* M. nitroreducens’ enrichment culture

All batch incubations in this study were seeded from an established enrichment culture of ‘*Ca*. M. nitroreducens’ (~30% of the community) performing nitrate-driven AOM. This system was seeded from a ‘*Ca.* M. nitroreducens’ enrichment established previously in our laboratory [3] that has operated under similar conditions for >10 years. Briefly, the parent system was a 2 l reactor vessel with 1.5 l of liquid and 0.5 l headspace fed weekly with methane (as a gas mix: 90% CH_4_, 5% CO_2,_ and 5% N_2_), nitrate (topped up to 14.2 mmol l^−1^) and ammonium (topped up to 14.2 mmol l^−1^).

### Batch experimentation

Batch incubations were set up in duplicate under two conditions: supplied either (1) arsenate and methane, or (2) nitrate and methane. Batch inoculum was prepared from a 300 ml aliquot of biomass suspension (at a protein concentration of 187.5 ± 4.1 μg/ml) from the parent bioreactor which was incubated in an anoxic vessel to consume the residual nitrate. Nitrate concentration was monitored with nitrate stripes (Merck) until depletion at ~8 days. In an anaerobic chamber (Coy), 30 ml of undiluted inoculum was dispensed into each 58 ml serum bottle. The As-AOM condition batches were amended with 0.5 mM of arsenate from a stock solution (50 mM Na_2_HAsO_4_·7H_2_O, Sigma-Aldrich). Simultaneously, the nitrate control treatment was amended with 5 mM of nitrate. All treatments were sealed with butyl rubber stoppers clamped with aluminium caps and then flushed with gas mix (95% Ar and 5% CO_2_) for 5 min prior to injection of 10 ml ^13^C-methane (99% atom ^13^C, Sigma-Aldrich). All batches were incubated on a magnetic stirrer (100 rpm) at 24 ± 2°C and incubated over 72 h.

### Geochemical sampling and measurements

Batch incubations were periodically sampled using an Ar-flushed 3-ml sterile syringe with 25-gague needle. Half of the cell suspension was filtered through a 0.22-μm syringe filter for chemical analysis. For As-amended batches, 150 μl of filtrate was used for measurement of As (III) with a KMnO_4_-reactive spectrophotometric method [13]. Residual filtrate was stored at −20°C for the measurement of N species (nitrate and ammonium) with a Lachat QuickChem8000 Flow Injection Analyzer (Lachat Instrument, Milwaukee, WI) and As speciation and accurate quantification by Inductively Coupled Plasma - Mass Spectrometry (ICP-MS) in the Environmental Analysis Laboratory at Southern Cross University (SCU). For ^13^CO_2_ quantification a 0.5 ml aqueous sample was immediately injected into a 3 ml helium flushed (for 5 mins) Exetainer vials (Labco) and preserved at -20°*C.* Prior to measurement, 0.2 ml 1 M HCl was injected into each thawed sample to extract CO_2_ which was analysed using gas chromatography–mass spectrometry (7890A/5975C; Agilent) and the ^13^CO_2_ ratio calculated as described previously [14].

### DNA and RNA extraction

Nucleic acids were extract from 2 ml cell suspension samples from each batch at 24 and 36 h. Half of the cell suspension was processed using a FastRNA Pro Soil-Direct Kit (MP Biomedicals, USA) for RNA extraction following the manufacturer’s protocol. RNA concentrations were quantified using the NanoDrop Spectrophotometer (Thermo Scientific). RNAseOUT (Invitrogen) was added to the extracted RNA sample to a concentration of 5% [v/v] before preservation at −80°C. The remaining biomass (1 ml) was used for DNA extraction using a FastDNA SPIN kit for soil (MP Biomedicals, USA) following the manufacturer’s protocol. DNA concentrations were quantified using the Qubit HS dsDNA assay kit (Invitrogen).

### DNA and RNA library preparation and sequencing

Paired-end libraries were prepared for all DNA extracts using a DNA Prep Kit (Illumina). The libraries were quantified using the Qubit dsDNA HS assay kit (Invitrogen) and sequenced by the Australian Centre for Ecogenomics (ACE) using a NovaSeq 6000 System (Illumina) to produce 2 Gb of shotgun metagenomic paired-end reads (2 × 150 bp). Metatranscriptomes were prepared from RNA extracted at the 36 h timepoints of all batches. RNA extracts were cleaned of residual DNA twice using the RNA Clean & Concentrator-5 kit (Zymo Research), including DNase I treatments. Following clean-up, RNA extracts were quantified using a Qubit HS RNA assay kit (Invitrogen) and RNA integrity was assessed using a TapeStation System (Agilent). Ribosomal-RNA depletion, cDNA generation, and library preparation were done by ACE using a Ribo-Zero Plus Kit (Illumina) following manufacturers protocol by ACE. RNA libraries were sequenced on a NovaSeq 6000 System (Illumina) generating ~9 Gb per 30 million paired-end reads (2 × 150 bp) per sample.

### Metagenomic analysis

#### Quality control, binning, and assembly of metagenomes

Quality of the returned reads was assessed using FastQC v0.11.9 (https://github.com/s-andrews/FastQC) and MultiQC v1.11, and reads were then trimmed using Cutadapt v3.5 [15]. Metagenomes were assembled using MetaSPAdes v3.11.1 [16]. MAGs were binned for each sample using Aviary v0.2.0 [17]. MAG quality was determined using CheckM2 v0.0.1 [18] and dereplicated at 95% average nucleotide identity using CoverM v0.6.1 [19] via the cluster function. MAGs with ≥70% completeness and ≤10% contamination were retained. These MAGs were dereplicated with closed MAGs recovered in a previous study from the same seed bioreactor using a combination of short- and long-read sequencing [20]. Microbial community profiles of each bioreactor batch were prepared by mapping paired-end reads to the retained MAGs using the CoverM v0.6.1 genome function. MAGs were assigned taxonomy using Genome Taxonomy Database (GTDB)-Tk v2.1.0 [21] which assigns GTDB release 07-RS207 taxonomy [22].

Replication rates of the ‘*Ca*. M. nitroreducens’ population at the 24 and 36 h timepoints were predicted by applying iRep v1.10 to metagenomic reads mapped to the closed ‘*Ca*. M. nitroreducens’ genome [23]. The returned metric, ‘bPTR’, refers to the ratio of read coverage between the origin and terminus of replication (which are identified based on GC-skew of the genome), where a value of 1 indicates no cells replicating, and 1.25 indicates 25% of cells replicating [23].

#### Functional annotation

Open reading frames (ORFs) were called for all MAGs using Prokka v1.14.6 [24]. ORFs were functionally annotated with KEGG Orthology (KO) [25] and PFAM [26] using EnrichM v0.6.3 (https://github.com/geronimp/enrichM) *via* the annotate function. Additional functional annotation was done using METABOLIC-G v4.0 [27]. Putative multiheme type *c* cytochromes (MHCs) were identified by searching for ORFs possessing ≥3 CXXCH motifs and further checking for cytochrome *c* domains via NCBI CD-Batch search [28, 29].

The presence of oxidoreductases that might mediate arsenate reduction in the reactor batches was investigated for all MAGs. Putative oxidoreductases were identified by screening for the molybdopterin oxidoreductase domain denoted by PFAM ID ‘PF00384’ [26]. The function of the molybdopterin oxidoreductase ORFs was inferred phylogenetically alongside a set of curated molybdopterin oxidoreductases retrieved from a previous publication [6]. First, the ORFs were aligned using Muscle v5.3 [30] with default parameters. A protein tree of the alignment was then constructed using IQ-Tree v3.0.1 [31] with bootstrapping of 1000 replicates and the LG + F + R9 model. The protein tree was visualized on iTOL [32].

### Metatranscriptomic analysis

#### Read mapping of mRNA and differential gene expression analysis

The metatranscriptomic paired-end reads were processed using the pipeline embedded in TranscriptM v.0.5 (https://github.com/sternp/transcriptm). To summarize, TranscriptM first assessed quality of reads using Kneaddata v0.7.7 (https://github.com/biobakery/kneaddata) and then trimmed reads using Trimmomatic v0.39 [33]. BAM files were generated by mapping trimmed reads to MAGs using CoverM v0.6.1 ‘Make’ and filtered via CoverM v0.6.1 ‘Filter’. DNA contamination was assessed based on the directionality of transcripts and removed from BAM files using the Bioconductor R package, StrandCheckR v1.15.0 [34]. Finally, raw count tables were generated using the Subread v2.0.3 package [35], and TPM values were calculated using the following equation:


$$ \mathrm{TPM}=\frac{\mathrm{rg}\times \mathrm{rl}\times{10}^6}{\mathrm{flg}\times T} $$

where rg = reads mapped to gene g, rl = read length, flg = feature length (or CDS length), and *T* = sum of rg^*^rl/flg for all genes. Differential gene expression analysis was performed for ‘*Ca*. M. nitroreducens’ using the Bioconductor R package, DESeq2 v3.15 [36], using the raw count table supplied by TranscriptM.

### Comparative genomics of ‘*Ca.* Methanoperedens’ *arrA* and *arxA*

#### Detection of arrA/arxA in ‘*Ca.* Methanoperedenaceae’ genomes

Publicly deposited ‘*Ca.* Methanoperedenaceae’ MAGs were identified on the GTDB Release 10-RS226 [22] and retrieved from NCBI Genbank Release 270 (February 2026) [37] to survey the prevalence of arsenate metabolizing genes within the family. The ‘*Ca*. M. nitroreducens’ putative *arrA* and *arxA* open reading frames (ORFs) were used as blast queries to identify homologous proteins in the additional ‘*Ca.* Methanoperedenaceae’ MAGs. Homologous proteins were determined via blastp with the following parameters: query cover ≥65%, evalue ≤1e^−10^, percent identity ≥25%. The putative reductase subunits and their homologues were subject to phylogenetic analysis alongside a set of curated molybdopterin oxidoreductase A-subunits from previous studies [6, 38]. Phylogenetic trees were generated by first aligning protein sequences using Muscle v5.3 [30] with the ‘super5’ parameter, trimming the alignment via trimAl 1.5.1 (https://github.com/inab/trimal) with the automated1 method, and phylogenetic placement was inferred using IQ-Tree v3.0.1 [31] ultrafast bootstrap approximation with 1000 replicates.

To identify potential sources of HGT, the final set of ‘*Ca.* Methanoperedenaceae’-ArrA/ArxA proteins were used as Diamond blastp [39] queries against all redundant protein coding regions published in GTDB Release 10-RS226, excluding those from ‘*Ca.* Methanoperedenaceae’ genomes (query cover ≥65%, evalue ≤1e^−10^, percent identity ≥25%, maximum targets = 15). A preliminary protein tree was generated with hits, and proteins most phylogenetically related to ‘*Ca.* Methanoperedenaceae’ were retained to generate a pruned tree. This pruned tree was visualized on iTOL v7 [32] alongside the untrimmed multiple sequence alignment (MSA) of the active site residues and formatted with Adobe Illustrator.

#### Comparison of arr/arx gene operons

The arrangement of the putative *arr* and *arx* gene clusters found within ‘*Ca.* Methanoperedenaceae’ genomes was compared to other organisms. Genomic regions surrounding the *arrA* and *arxA* ORFs were extracted and protein coding regions were predicted with Prodigal v2.6.3 [40] via Prokka v1.15.6 [24]. To identify highly conserved genomic regions, a pairwise alignment was performed using minimap2 v2.30 [41] with the following parameters: ‘minimap2 -X -p 0.1’. The R-packages gggenomes v1.1.3 [42] and ggtree v1.14.6 [43] were used to visualize the gene clusters, ordered by ArrA/ArxA phylogeny. Gene annotations were inferred using the Prokka annotations, operon structures described in previous studies [10, 38, 44, 45], and protein homology.

To further explore the HGT acquisition site for arrA/arxA, gene synteny analysis was done for larger genomic regions (up to 60 kb) between ‘*Ca.* Methanoperedens’ species. Contigs containing the *arr/arx* gene cluster were annotated using BAKTA v1.11.3 [46], then the BAKTA output was used for gene cluster analysis via the clinker v0.0.32 pipeline [47]. Gene synteny where amino acid identity was >30% was visualized using the CAGECAT interface [48], and annotations inferred from Prokka and BAKTA annotations, known operon structures, and protein homology.

## Results and discussion

### Incongruent Arr/Arx distribution in '*Ca.* Methanoperedenaceae'

The prevalence of arsenate reductase (*arr*) and arsenite oxidoreductase (*arx*) gene clusters across the ‘*Ca.* Methanoperedenaceae’ was assessed by screening publicly available MAGs representing the family. High-quality (>70% complete, <10% contamination) MAGs were screened for ArrA and ArxA based on protein phylogeny to functionally characterized reference sequences. A total of 51 high-quality MAGs representing the family were identified, with 11 of these encoding either *arr* or *arx,* or both (Fig. 1, Supplementary Table S1). These MAGs, representing arsenate metabolizing species, were recovered from diverse and globally distributed environmental sites including a deep underground gold mine site [49], a terrestrial mud volcano [50], various arsenic contaminated sediments [6], and from sediment-seeded methane-fed bioreactors [3, 5, 51] (Fig. 1, Supplementary Table S1). The distributions of *arr* and *arx* do not align with MAG phylogeny (Fig. 1), as noted generally for respiratory oxidoreductases encoded by members of the family [6], consistent with gene retention or loss driven by electron-acceptor availability. Gene synteny analysis across the Arr/Arx-encoding ‘*Ca.* Methanoperedenaceae’ indicated dissimilar genomic contexts, even between more closely related species ‘*Ca*. M. nitroreducens’ and BE2012, suggesting that sites of gene acquisition within the family are not conserved. ‘*Ca.* M. nitroreducens’ and BE2012 encoded both *arr* and *arx* in tandem within maintained gene clusters (Supplementary Fig. S1). The syntenic Arx/Arr gene cluster region between these two species (~40 kb) included other genes, such as DNA ligase and RNA helicase that were likely inherited from the same HGT event. Some MAGs encoded genes indicative of genomic rearrangement and/or HGT events (eg, IS66 family transposase, toxin-antitoxin system, and recombinases) within the vicinity of the Arr/Arx gene cluster [52] (Supplementary Fig. S1). These genomic contexts suggest flexible genomic arrangement within the ‘*Ca.* Methanoperedenaceae’ family for incorporation of foreign respiratory gene clusters [52, 53].

**Figure 1:**
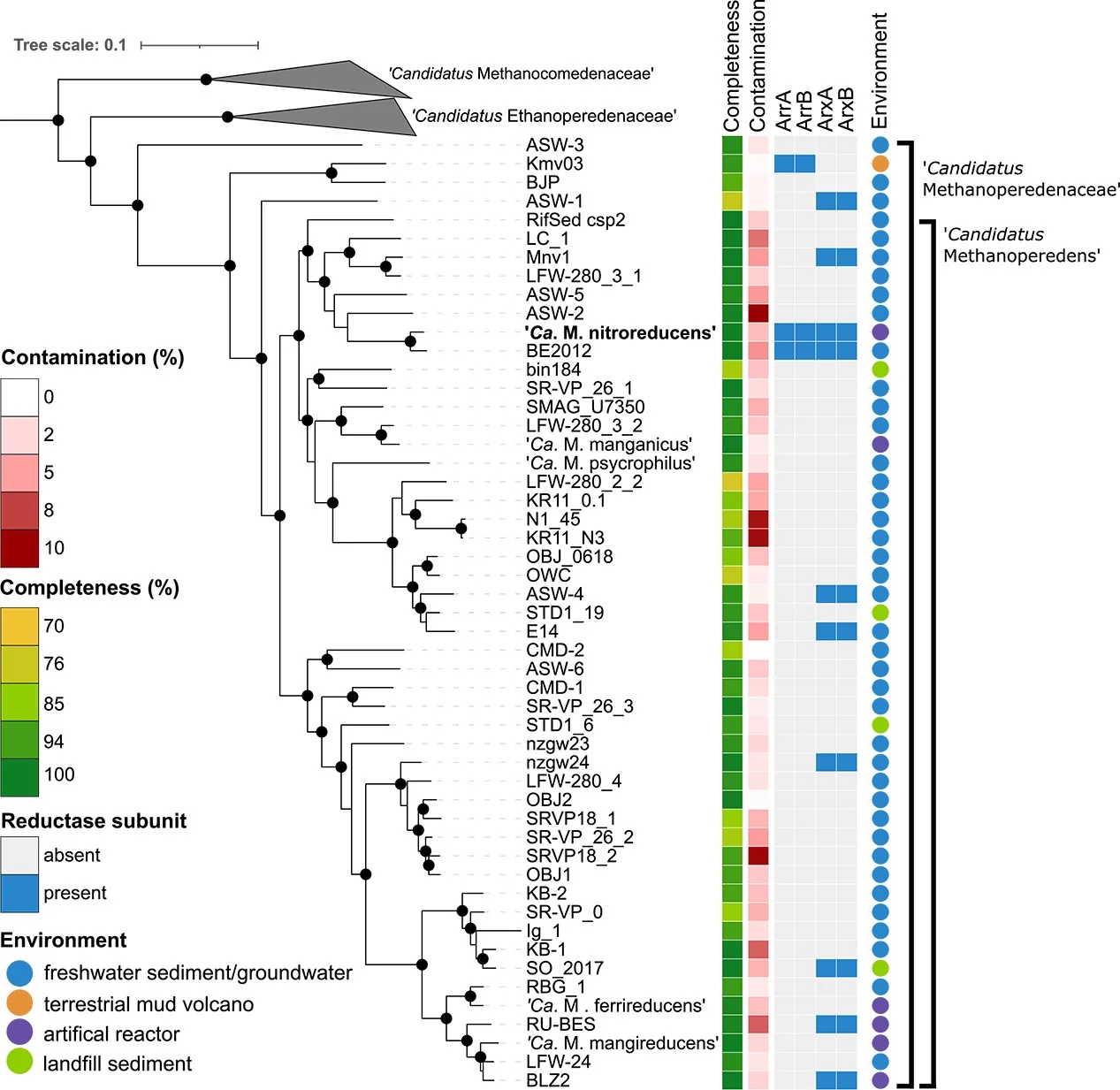
Phylogenetic placement of ‘*Ca.* Methanoperedenaceae’ metagenome assembled genomes (MAGs) and distribution of Arr and Arx catalytic subunits across lineage. MAGs were retrieved from the Genome Taxonomy Database (GTDB) release 226. MAG completeness and contamination were predicted by CheckM2 (see methods), and environment indicates the isolation source of the MAG according to associated publications or NCBI BioProject metadata. The genome tree was inferred using maximum-likelihood with a concatenated set of 122 archaeal specific marker genes. Black circles represent bootstrap support >85% with 1000 replicates. ‘*Ca*. M. nitroreducens’, explored in the current study, is emphasized in bold text.

Blastp analysis indicated that Arr and Arx encoded by ‘*Ca.* Methanoperedens’ are most closely related (up to ~76% AAI) to those found in bacteria rather than related archaeal species, suggesting the genes were inherited horizontally from phylogenetically distant organisms (Supplementary Table S2). Phylogenetic placement and gene synteny analysis suggest three separate HGT events, with Methanoperedens-ArrA forming two separate clades (Fig. 2, Supplementary Fig. S2). The closest homologue of Kmv03-ArrA belonged to an uncharacterized MAG of the Dehalococcoidales order (76.5% identity, Supplementary Table S2) and ANME-2ab (*Ca*. ‘Methanocomedenaceae’, 76.1%, Supplementary Table S2). The ‘*Ca*. M. nitroreducens’ ArrA was found most homologous to a protein encoded by a separate order within the Dehalococcoidia class (63.9% identity, Supplementary Table S2). For Methanoperedens-Arx, the closest homologue was from an uncharacterized Bacillota MAG (56% identity, Supplementary Table S2).

**Figure 2:**
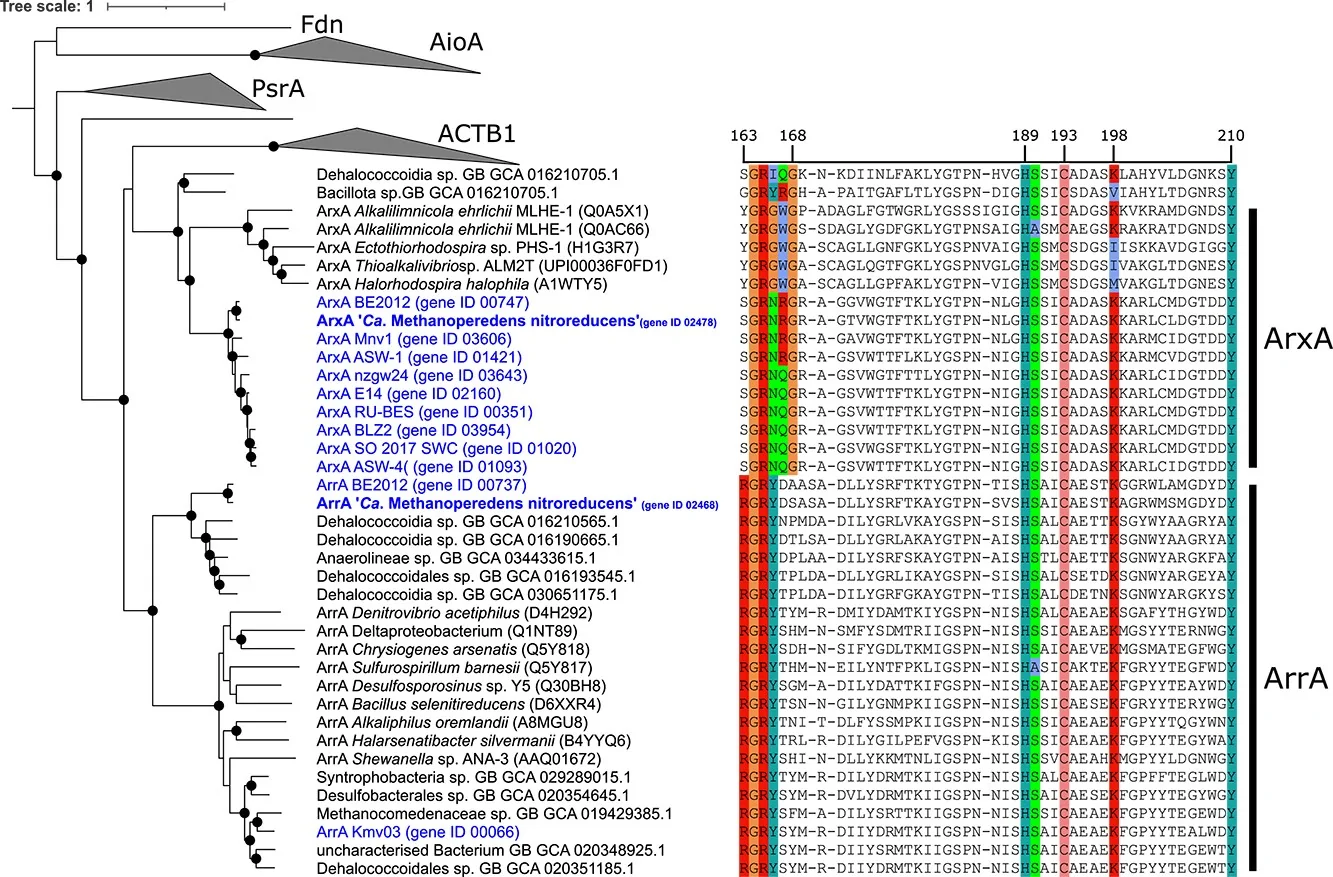
Phylogenetic inference of arsenate reductase (Arr) and arsenite oxidase (Arx) subunit A alongside multiple sequence alignment of predicted active side residues. Representative ArrA/ArxA proteins are included, as well as blastp hits (see methods, Supplementary Tables S2 and S6) derived from MAGs deposited in the Genome Taxonomy Database, with Genbank accessions indicated in brackets. ‘*Ca*. M. nitroreducens’ is emphasized in bold text. The dendrogram represents a protein tree based on alignment of the A-subunit of the *arr* and *arx*, inferred by maximum likelihood, and support values were calculated via nonparametric bootstrapping with 1000 replicates. Black dots indicate bootstrap support >85% (1000 replicates). Active site residues are indicated by shading, with the ClustalW colour scheme and annotations according to a previous study [10]. Residue positions are annotated to be consistent with *Shewanella* ANA-3 ArrA, as per the previous study. Canonical bacterial ArrA enzymes harbour a RGRY motif (residues 163–166); bacterial ArxA sequences contain a GRGWG motif (residues 164–168), whereas ‘*Ca.* Methanoperedenaceae’-Arx contain a GRN(R/Q)G motif in the same position. Other active-site residues (described in the previous study) were generally conserved across all enzymes (residues 189, 193, 198, 210) [10].

ArrA and ArxA are reported to have distinct, conserved active site motifs which are involved in substrate binding and suspected to underpin reaction directionality [10]. We found that the active site motif of ‘*Ca.* Methanoperedens’-ArrA was consistent with bacterial homologues (RGRY, Fig. 2). The predicted active site motif for ‘*Ca.* Methanoperedens’-ArxA, SGRN(Q/R)G [10], was conserved within the family, but is distinct from the motif conserved in experimentally characterized bacterial homologues, XGRGWG (Fig. 2). In addition, it was previously reported that Arx distribution amongst bacteria is correlated with the ubiquinone (*ubi*) biosynthetic pathway whereas Arr distribution is correlated with the futalosine/menaquinone (*mqn/men*) biosynthetic pathway, likely due to thermodynamic constraints of these quinone/quinol partners [11]. Given that ‘*Ca.* Methanoperedenaceae’ encode the futalosine pathway [54] and that their Arx active-site motif deviates from characterized bacterial homologues, their Arx may not function as a canonical arsenite oxidoreductase.

The *arx* and *arr* gene clusters in ‘*Ca.* Methanoperedenaceae’ contained all required subunits (*arr/arx*ABCD) for expression of the mature Arr/Arx complex as commonly observed in bacteria (Supplementary Fig. S2) [10, 38]. This includes a predicted NrfD-like transmembrane subunit, Arr/ArxC, thought to transfer electrons to or from the quinone pool, and Arr/ArxD, a TorD-like cytoplasmic chaperone predicted to facilitate active oxidoreductase maturation and/or translocation into the periplasm directed by the twin-arginine translocation system [10, 55, 56]. Also within the gene cluster are a putative response regulator (Arr/ArxR), a sensor histidine kinase, and periplasmic binding protein (Arr/ArxX) [57]. The gene cluster arrangement and direction of the core *arx* genes were also homologous to those observed in bacterial representatives. ‘*Ca.* Methanoperedenaceae’ core *arr* gene clusters were conserved as *arrABCD*; however, bacterial gene clusters often excluded *arrC* or were arranged as *arrABDC*. Some ‘*Ca.* Methanoperedenaceae’ MAGs were found to encode an archaeal *tatA* directly upstream of the *arrA/arxA*-subunit, which mediates the translocation of fully folded proteins encoding a Tat-signal across the pseudoperiplasmic membrane [58]. These genes may be indicative of an adaptation of the species cell architecture to accommodate horizontally inherited bacterial gene clusters [53].

### ‘*Ca*. M. nitroreducens’ enrichment culture mediates As-AOM

The genome-predicted ability of ‘*Ca.* M. nitroreducens’ to mediate As-AOM was also determined using biomass from a long-term enrichment culture performing nitrate-driven AOM [3] and consistently dominated (~30%) by a strain known to encode *arrAB* and *arxAB* [6]. When the enrichment culture biomass was incubated with methane and arsenate (As(V)), the latter was reduced to arsenite (As(III)) within 72 h with a nearly complete conversion (Fig. 3a, Supplementary Fig. S3). Active AOM was observed with arsenate amendment, as evidenced by the steady increase in concentration of ^13^CO_2_ produced from ^13^CH_4_ oxidation (Fig. 3a), a trajectory analogous to that of canonical nitrate-driven AOM by ‘*Ca.* M. nitroreducens’ (Fig. 3b). The ^13^CO_2_ accumulation rate linked to AOM was 2.0 ± 0.1 μmol h^−1^ in the arsenate-amended incubation (Fig. 3c); about 9 times lower than in the nitrate-amended incubation (18.5 ± 0.5 μmol h^−1^) (Fig. 3b). Kinetic fitting demonstrated near-identical rates of As(V) reduction (8.1 ± 0.2 μmol h^−1^) and As(III) production (7.4 ± 0.2 μmol h^−1^). The close correlation between these rates and that of AOM thereby supports a ~4:1 stoichiometry (Fig. 3d), consistent with As-AOM (Eq. 1) [59]:


(1) \begin{align*} 4{\mathrm{H}\mathrm{AsO}}_4^{2-}+{\mathrm{CH}}_4+7{\mathrm{H}}^{+}\to 4{\mathrm{H}}_3{\mathrm{AsO}}_3+{\mathrm{H}\mathrm{CO}}_3^{-}\notag\\+{\mathrm{H}}_2\mathrm{O}\Delta \mathrm{G}{{}^{\circ}}^{\prime }=-478.2\ \mathrm{kJ}\ {\mathrm{mol}}^{-1}\ \mathrm{CH}4 \end{align*}

**Figure 3:**
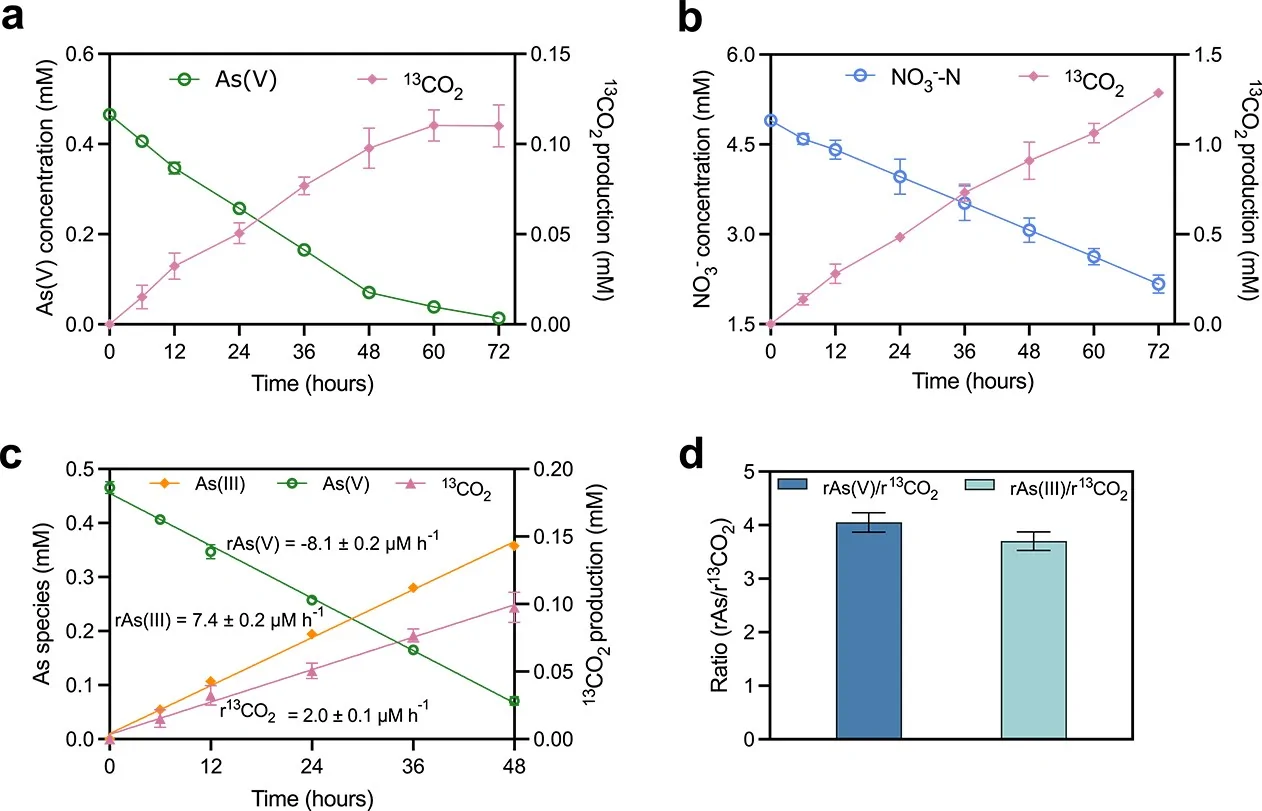
Coupled ^13^CH_4_ oxidation (inferred from ^13^CO_2_ production) and As(V) reduction in batch incubations by ‘*Ca*. M. nitroreducens’ enrichment culture. Error bars indicate standard deviations of two independent biological replicates. (a) Monitoring of As(V), and ^13^CO_2_ accumulation during the operation of the arsenate-fed batch. (b) Monitoring of NO_3_^−^ concentration and ^13^CO_2_ accumulation during nitrate-fed batch operation. Labelled ^13^C was supplied at time 0 as ^13^CH_4_. (c) Profiles and rates of As(V) reduction, As(III) production, and ^13^CO_2_ production during incubation with As(V) and ^13^CH_4_. Rates are determined by linear regression from the respective measured concentrations (during the period of 0 to 48 h). The confidence level for fitting curves was 95%. (d) Ratios of As(V) reduction and As(III) production rates, respectively, to the AOM rate (^13^CO_2_ production rate) in the incubation with As(V) and ^13^CH_4_.

These observations collectively support the coupling of arsenate reduction to AOM by the ‘*Ca*. M. nitroreducens’ enrichment culture. The enriched culture reduced arsenate at a rate of 1.44 ± 0.04 μmol As(V) h^−1^ mg protein^−1^, which falls within the range reported for whole-cell arsenate-respiring microorganisms (~0.1–10 μmol As(V) h^−1^ mg protein^−1^), such as well-characterized arsenate reducers such as *Geobacter* sp. OR-1 and *Shewanella* sp. ANA-3 [60, 61].

### Composition and metabolic activity of mixed microbial community supports As-AOM mediated by ‘*Ca*. M. nitroreducens’

Metagenomic analysis of the bioreactor batches recovered 24 high quality (> 70% complete, <10% contamination) MAGs (Supplementary Table S3). The final MAG set represented ~87% of the metagenome and ~ 89% of the total metatranscriptome (Fig. 4a, Supplementary Table S3). Both the nitrate and arsenate-fed conditions were dominated by ‘*Ca.* M. nitroreducens’ (MAG-ID: Methanoperedens) in the metagenome (average ~ 31%) and metatranscriptome (average ~ 80%). Other abundant organisms included unclassified species of the class Bacteroidota (~23% relative abundance; MAG-ID Bacteroidota-1) and the family *Melioribacteraceae* (~15% relative abundance; MAG-ID Melioribacteriaceae-1), although each made up <2% of the total transcriptome, indicating a relatively low activity. The second most active population based on the metatranscriptome was annamox bacterium ‘*Candidatus* Kuenenia stuttgartiensis’ (MAG-ID Kuenenia), which accounted for ~6% and ~3% of the total TPM for the arsenate and nitrate-fed batches, respectively. Inferred replication rates of the ‘*Ca.* M. nitroreducens’ population, based on the ratio of metagenomic sequencing coverage between the origin and terminus of replication [23], indicated a similar growth rate for the species when arsenate replaced nitrate, suggesting the observed As-AOM could also support growth of the species (Supplementary Fig. S4).

**Figure 4:**
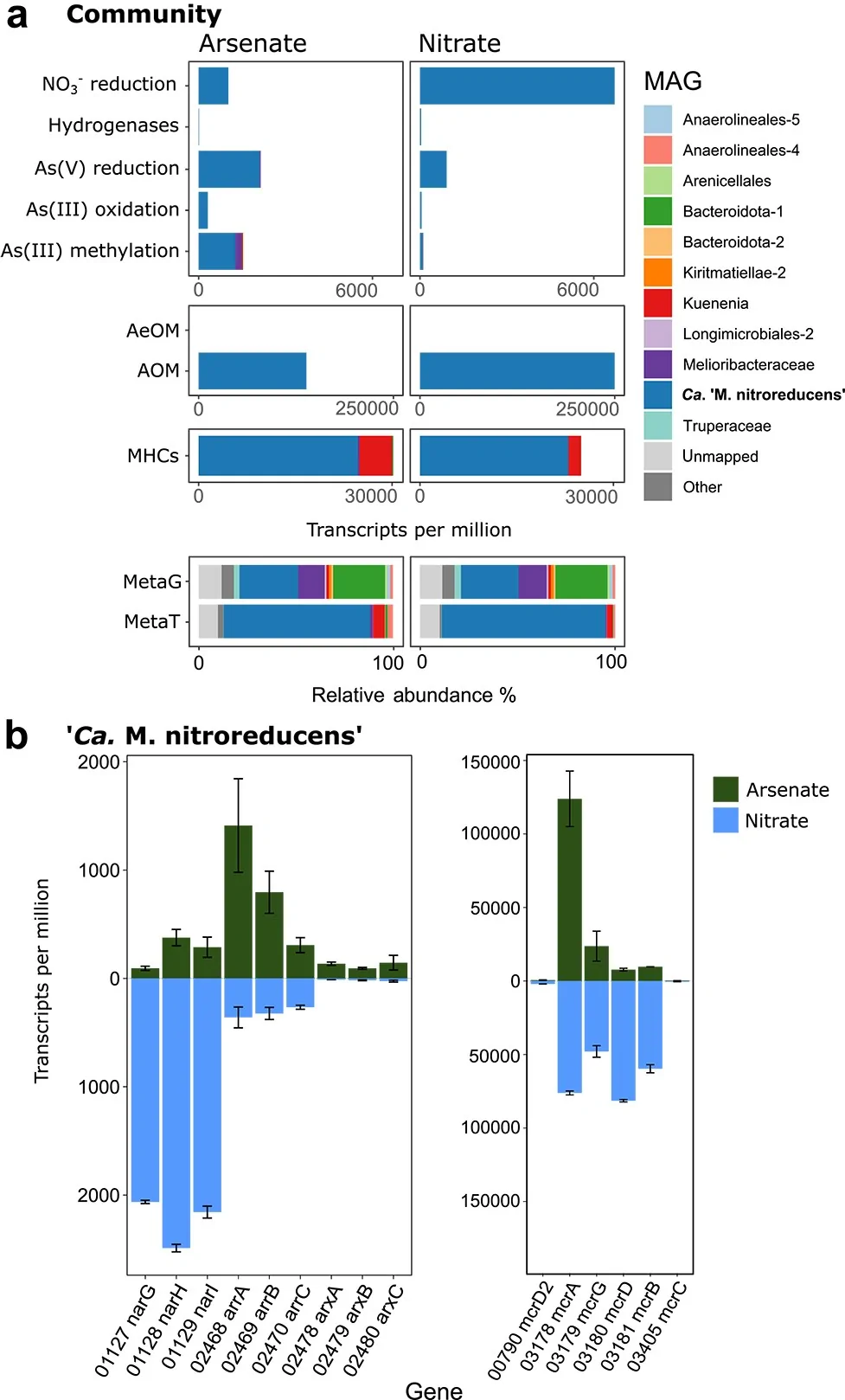
Microbial community composition and transcriptional activity under arsenate-reducing vs nitrate-reducing conditions. Transcripts per million (TPM) and relative abundances are shown as the average of two biological replicates. (a) Metagenomic (MetaG) and metatranscriptomic (MetaT) profile of the microbial community abundant organisms and key pathways from arsenate-fed versus nitrate-fed anaerobic methane oxidizing batch incubations at 36 h. AeOM, aerobic methane oxidation; AOM, anaerobic oxidation of methane; MHCs, multiheme type c cytochromes. (b) Differential expression of key terminal oxidoreductase complexes by ‘*Ca*. Methanoperedens nitroreducens’ under arsenate-fed versus nitrate-fed anaerobic methane oxidizing batch incubations at 36 h. Error bars indicate standard deviation.

The bioreactor derived MAGs were functionally annotated to identify microbial populations contributing to As-AOM. ‘*Ca.* M. nitroreducens’ was the only population encoding a methane-oxidation pathway, with the methyl-coenzyme M reductase complex (*mcrAGDBC*) highly transcribed, contributing to ~25% of the nitrate-fed batch metatranscriptome, and ~15% of the arsenate-fed metatranscriptome (Fig. 4a, Supplementary Table S4). Reductase complexes involved in arsenic-respiration were investigated by screening the MAGs for molybdoenzyme oxidoreductase A-subunits. ‘*Ca.* M. nitroreducens’ was the only population to encode oxidoreductases homologous to *arrA* and *arxA* (Supplementary Fig. S5). MAGs encoding the aerobic arsenite oxidase complex (Aio) were also not detected in the bioreactor batches. For ‘*Ca.* M. nitroreducens’, 17 of the 38 encoded MHCs, typically implicated in adaptive extracellular electron pathways for the organism [1, 60], were significantly upregulated (*P*_adj_ < 0.005; log2fold change >1, Supplementary Table S5) under As-AOM conditions. Some of these MHCs might be involved in connecting the oxidoreductase complex to the quinone pool, as was suggested previously suggested for the nitrate reductase NarGH of ‘*Ca.* M. nitroreducens’ sp. MPEBLZ [61]. As-AOM by MHC-mediated extracellular electron transfer to syntrophic partners has been previously hypothesized [59]; however, the absence of bacterial arsenate reductases in our system does not support this hypothesis here. Further characterization of the differentially expressed MHCs is required to determine their potential role in As-AOM.

### Transcriptional response of ‘*Ca.* M. nitroreducens’ to As-AOM conditions suggests an adaptive metabolic strategy *via* co-opted bacterial oxidoreductase complexes

The transcriptional response of ‘*Ca.* M. nitroreducens’ to As-AOM compared to nitrate-AOM was examined in more detail. In total, 1205 genes encoded by ‘*Ca.* M. nitroreducens’ were differentially expressed (*P*_adj_ ≤ 0.05) (Supplementary Table S5). Under arsenate-fed conditions, genes predicted to be involved in arsenic detoxification, stress–response, and motility were highly upregulated by ‘*Ca*. M. nitroreducens’, potentially due to the toxicity of the added arsenate and accumulated arsenite (Supplementary Table S5). This included expression of an arsenite methyltransferase (log_2_FoldChange = 4.86, Fig. 4a) suggesting that some arsenic methylation may have occurred. However, any such activity appears to have been minor, as evidenced by the near-stoichiometric recovery of arsenic as inorganic As(V) and As(III) throughout the experiment (Supplementary Fig. 2). These stress responses could be an explanation for the observed decrease in AOM rate during the As-amended condition (Fig. 4).

Both the putative *arr* (A-subunit mean TPM: As-AOM = 1411; n-AOM = 359) and *arx* (A-subunit mean TPM: As-AOM = 136; n-AOM = 12) complexes were significantly upregulated by ‘*Ca*. M. nitroreducens’ in the arsenate-fed batch, whilst the nitrate reductase (*narGHI*) was significantly downregulated (G-subunit mean TPM: As-AOM = 93; n-AOM = 2064; Fig. 4b, Supplementary Table S5). *ArxAB* was the most significantly differentially expressed oxidoreductase (*P*_adj_ = 1.21e^−53^, log_2_FoldChange = 4.6), supporting its importance for ‘*Ca.* M. nitroreducens’ under As-AOM conditions (Fig. 4b, Supplementary Table S5). While the Arr and Arx complexes were originally thought to catalyse only arsenate reduction [62, 63] or arsenite oxidation [38, 44, 45], respectively, more recent research has shown both can operate bidirectionally [11]. Since both are upregulated for the ‘*Ca.* M. nitroreducens’ population under As-AOM conditions, it is possible that both complexes can mediate arsenate reduction for the species (Fig. 4b).

## Conclusion

Collectively, this study supports the ability of ‘*Ca.* M. nitroreducens’ to independently redirect methane-derived electrons to arsenate via HGT-inherited oxidoreductase complexes. The occurrence of Arr and Arx oxidoreductases encoding ‘*Ca.* Methanoperedenaceae’ from globally distributed sampling sites suggests that arsenate may represent a widespread methane sink. This study provides direct evidence for the microbial mechanism responsible for As-AOM, further highlighting the remarkable genetic flexibility of the ‘*Ca.* Methanoperedenaceae’, expanding their known roles in linking methane cycling with diverse geochemical cycles. We extend current understanding of the role of ‘*Ca.* Methanoperedenaceae’ in environmental arsenate cycling and mobilization, building on evidence that iron-reducing members of the family mediate the release of As(V) bound to iron oxides in sediments via reductive dissolution [7, 8]. Microbial processes such as these are collectively the primary cause of arsenic leeching from sediment into natural water reservoirs leading to toxic concentrations [64, 65]. Understanding the natural processes that underpin arsenic poisoning, greenhouse gas release, as well as links between them, is crucial to resolving two significant global health burdens.

## Supplementary Material

Supplementary_material_wrag251

## Data Availability

Metagenomic and metatranscriptomic reads can be accessed under BioProject ID PRJNA1475091 from the NCBI database. Data tables generated from computational analyses in this study are provided in Supplementary Tables S1–S6.
