# Supplementary material for: Arsenate reduction coupled to anaerobic oxidation of methane by members of the Methanoperedenaceae
Source: ISME J. 2026 Sep 26;20(1):wrag251. doi: 10.1093/ismejo/wrag251 (PMC13685797; doi:10.1093/ismejo/wrag251)
Supplement: Supplementary file 1 [file supplementary_material_wrag251.zip › Figure_S4.pdf]

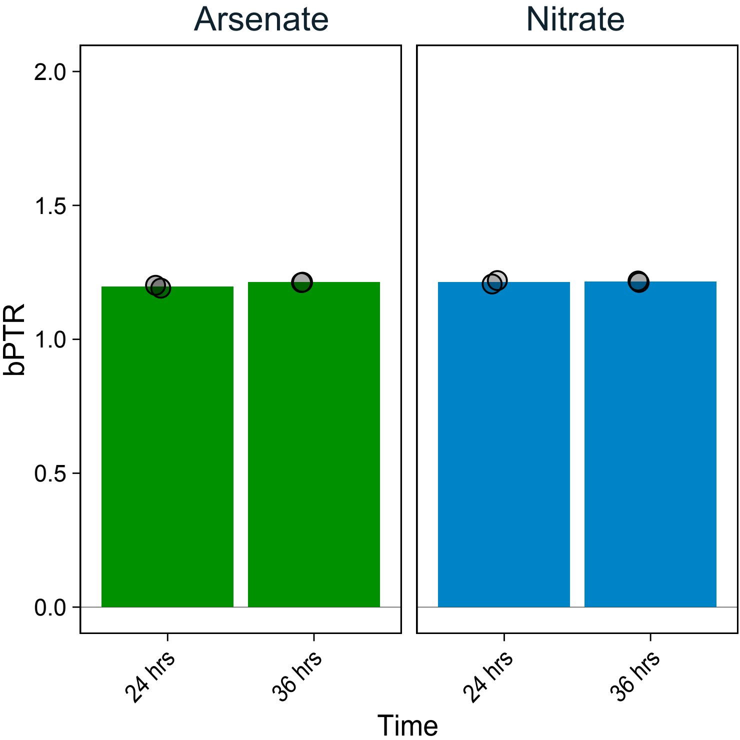

Arsenate
Nitrate
bPTR
2.0
1.5
1.0
0.5
0.0
24 hrs
36 hrs
24 hrs
36 hrs
Time
