# Supplementary material for: Arsenate reduction coupled to anaerobic oxidation of methane by members of the Methanoperedenaceae
Source: ISME J. 2026 Sep 26;20(1):wrag251. doi: 10.1093/ismejo/wrag251 (PMC13685797; doi:10.1093/ismejo/wrag251)
Supplement: Supplementary file 1 [file supplementary_material_wrag251.zip › Figure_S5.pdf]

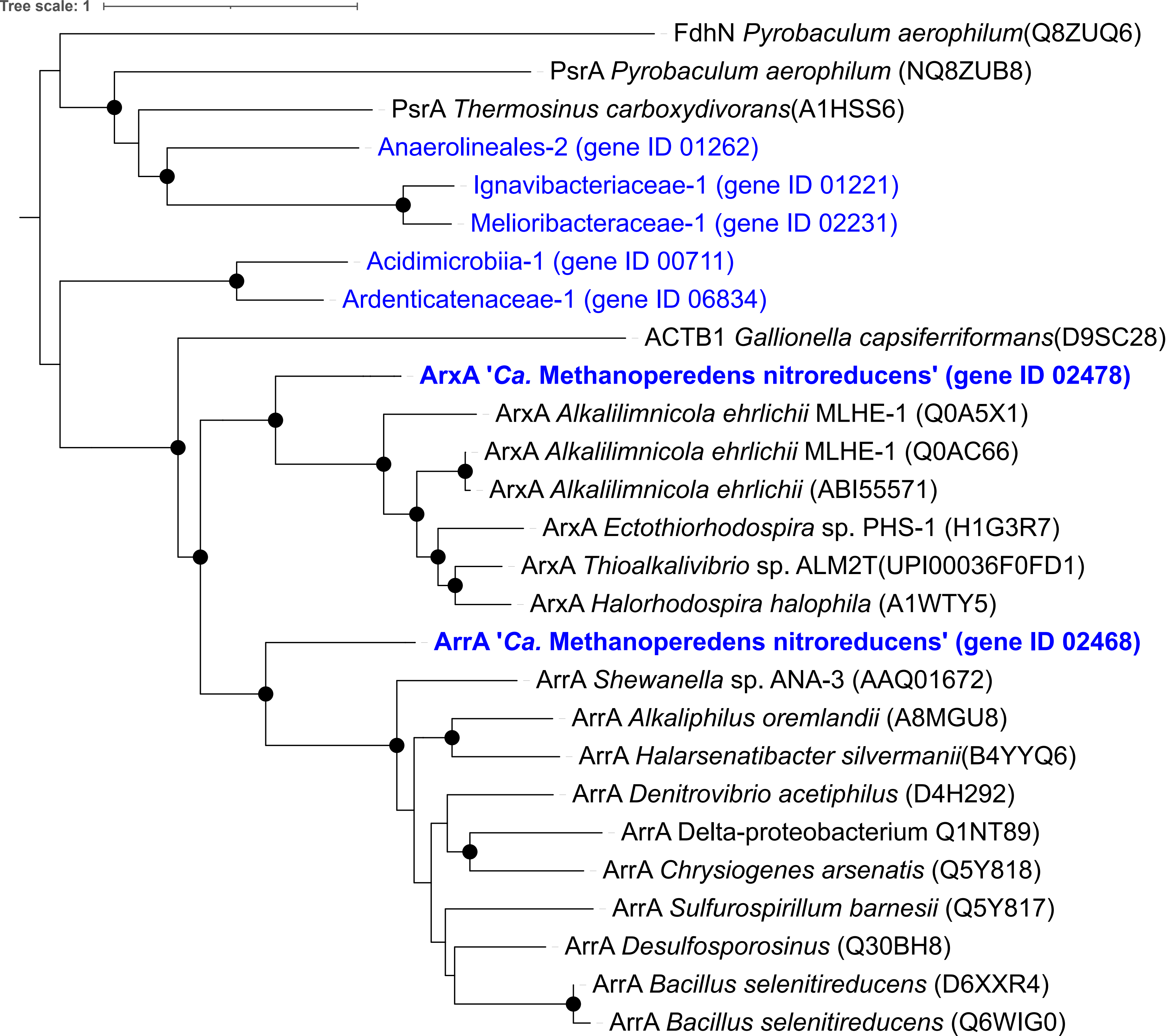
Tree scale: 1
FdhN Pyrobaculum aerophilum(Q8ZUQ6)
PsrA Pyrobaculum aerophilum (NQ8ZUB8)
PsrA Thermosinus carboxydivorans(A1HSS6)
Anaerolineales-2 (gene ID 01262)
Ignavibacteriaceae-1 (gene ID 01221)
Melioribacteraceae-1 (gene ID 02231)
Acidimicrobiia-1 (gene ID 00711)
Ardenticatenaceae-1 (gene ID 06834)
ACTB1 Gallionella capsiferriformans(D9SC28)
ArxA 'Ca. Methanoperedens nitroreducens' (gene ID 02478)
ArxA Alkalilimnicola ehrlichii MLHE-1 (Q0A5X1)
ArxA Alkalilimnicola ehrlichii MLHE-1 (Q0AC66)
ArxA Alkalilimnicola ehrlichii (ABI55571)
ArxA Ectothiorhodospira sp. PHS-1 (H1G3R7)
ArxA Thioalkalivibrio sp. ALM2T(UPI00036F0FD1)
ArxA Halorhodospira halophila (A1WTY5)
ArrA 'Ca. Methanoperedens nitroreducens' (gene ID 02468)
ArrA Shewanella sp. ANA-3 (AAQ01672)
ArrA Alkaliphilus oremlandii (A8MGU8)
ArrA Halarsenatibacter silvermanii(B4YYQ6)
ArrA Denitrovibrio acetiphilus (D4H292)
ArrA Delta-proteobacterium Q1NT89)
ArrA Chrysiogenes arsenatis (Q5Y818)
ArrA Sulfurospirillum barnesii (Q5Y817)
ArrA Desulfosporosinus (Q30BH8)
ArrA Bacillus selenitireducens (D6XXR4)
ArrA Bacillus selenitireducens (Q6WIG0)
