# Supplementary material for: Arsenate reduction coupled to anaerobic oxidation of methane by members of the Methanoperedenaceae
Source: ISME J. 2026 Sep 26;20(1):wrag251. doi: 10.1093/ismejo/wrag251 (PMC13685797; doi:10.1093/ismejo/wrag251)
Supplement: Supplementary file 1 [file supplementary_material_wrag251.zip › Supplementary_final.docx]

**Arsenate reduction coupled to anaerobic oxidation of methane by members of the *Methanoperedenaceae***

Georgina H. Joyce^a,¥,φ^, Xueqin Zhang^b,φ^, Andy O. Leu^a^, Yosephine Gumulya^a,€^, Jianhua Guo^b^, Gene W. Tyson^a^, Shihu Hu^b,*^, Simon J. McIlroy^a,*^

^a^ Centre for Microbiome Research, School of Biomedical Sciences, Queensland University of Technology, Translational Research Institute, Brisbane, Australia

^b^ Australian Centre for Water and Environmental Biotechnology, The University of Queensland, Brisbane, Australia

^¥^ Present address: School of Chemistry and Molecular Biosciences, The University of Queensland, Brisbane, Australia

^€^ Present address: School of Agriculture and Food Sustainability, The University of Queensland, Brisbane, Australia

^φ^ These authors contributed equally

**Supplementary Information**

**Supplementary Figure legends**

**Figure S1:** Arrangement of *arr* and *arx* gene clusters found across ‘*Ca*. Methanoperedenaceae’ MAGs (red) compared to other prokaryotes. The dendrogram represents a structure-informed protein tree based on alignment of the A-subunit of the *arr* and *arx*, inferred by maximum likelihood, and support values were calculated via nonparametric bootstrapping with 1000 replicates. Gene functional predictions are noted by colour (“conserved genes”), with other genes including hypothetical genes coloured light grey. Pairwise nucleotide synteny between adjacent leaves is displayed in light yellow, as determined using minimap2 (see methods). Leaves are named as per GTDB taxonomy, with full taxonomies and genbank/refseq accessions provided in supplementary dataset S7. Asterisks (adjacent to MAG BE2012 and ‘*Ca.* M. nitroreducens’) indicate contiguity of the genome sequence, with the sequence continuing at the genome’s corresponding asterisk.

**Figure S2:** Gene synteny analysis of ‘*Ca*. Methanoperedenaceae *arr* and *arx* genomic contexts. Homologous genes are indicated by colour, with the core *arr/arx* genes displayed in the legend, and other annotations displayed adjacent to the gene. Genomes are arranged by phylogenetic placement (see In-text Figure 1). Synteny > 30% AAI between adjacently displayed genomes are indicated by greyscale connections. Where available, Genbank identifiers are displayed beneath the genome ID. Illustration made using the CAGECAT platform [1].

**Figure S3:** Monitoring of As(V) and As(III) during the operation of the arsenate-fed anaerobic methane oxidising batches. Error bars represent the standard deviation of two biological replicates.

**Figure S4:** Predicted replication rates (‘bPTR’) of the ‘*Ca*. M. nitroreducens’ population at the 24 hr and 36 hr timepoints of nitrate versus arsenate fed AOM batches. ‘bPTR’, refers to the ratio of read coverage between the origin and terminus of replication (which are identified based on GC-skew of the genome), where a value of 1 indicates no cells replicating, and 1.25 indicates 25% of cells replicating. Individual values of the duplicate metagenomic samples are displayed as grey circles. See previous study for further details on bPTR calculation [2].

**Figure S5:** Phylogenetic placement of complex iron-sulfur molybdoenzyme oxidoreductase A-subunits encoded by MAGs in As-AOM batches (blue text). Black text = reference proteins, with Uniprot/Uniparc ID in brackets. Bootstrap support > 85% (1000 replicates) is indicated by a black circle.

1.	Belt M van den, Gilchrist C, Booth TJ, Chooi YH, Medema MH, Alanjary M. CAGECAT: The CompArative GEne Cluster Analysis Toolbox for rapid search and visualisation of homologous gene clusters. *BMC Bioinformatics* 2023;**24**(1):181. <https://doi.org/10.1186/s12859-023-05311-2>

2.	Brown CT, Olm MR, Thomas BC, Banfield JF. Measurement of bacterial replication rates in microbial communities. *Nat Biotechnol* 2016;**34**(12):1256–63. <https://doi.org/10.1038/nbt.3704>
