# Supplementary material for: Arsenate reduction coupled to anaerobic oxidation of methane by members of the Methanoperedenaceae
Source: ISME J. 2026 Sep 26;20(1):wrag251. doi: 10.1093/ismejo/wrag251 (PMC13685797; doi:10.1093/ismejo/wrag251)
Supplement: Supplementary file 1 [file supplementary_material_wrag251.zip › Figure_S1.pdf]

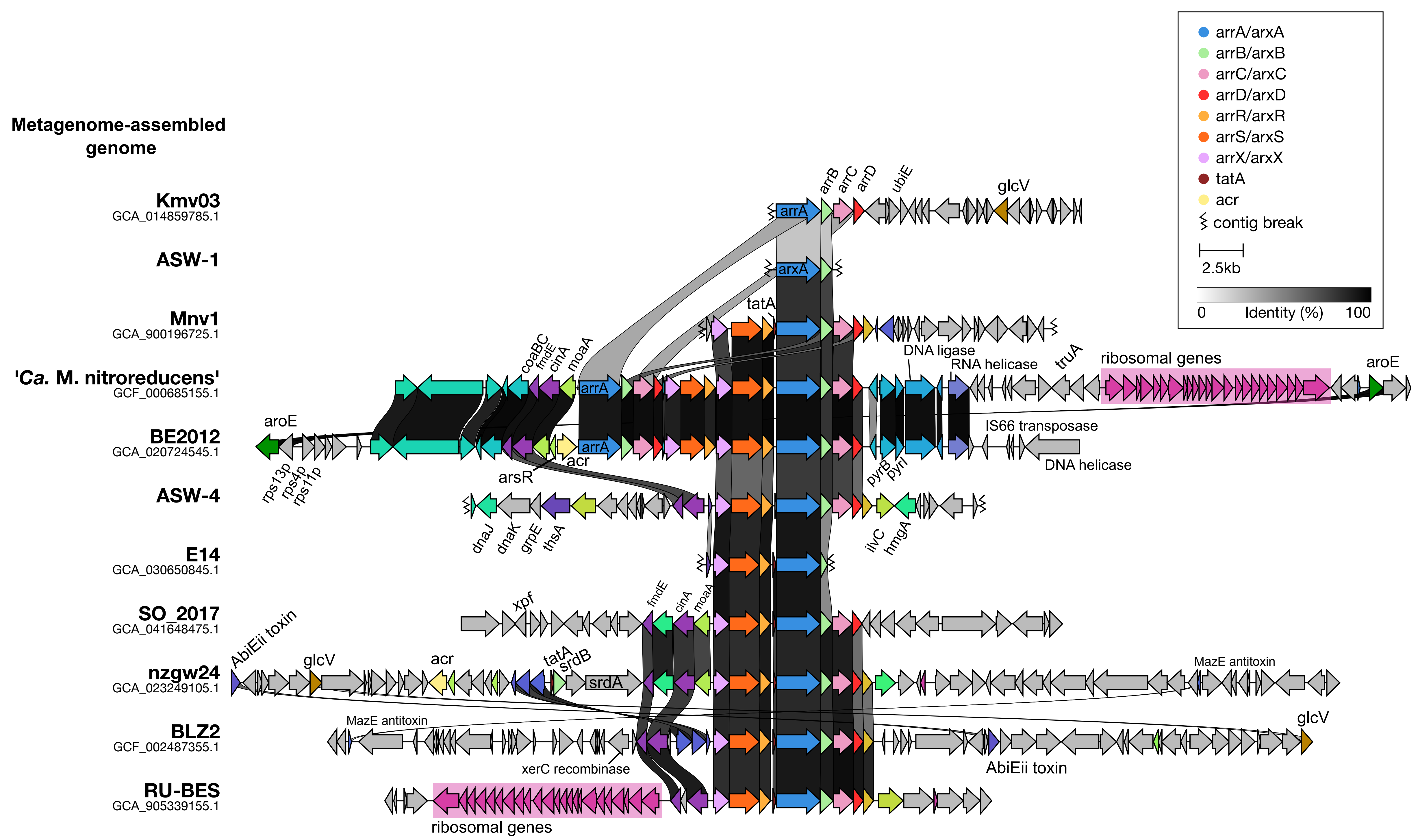

Metagenome-assembled genome
Kmv03
GCA_014859785.1
ASW-1
Mnv1
GCA_900196725.1
'Ca. M. nitroreducens'
GCF_000685155.1
BE2012
GCA_020724545.1
ASW-4
E14
GCA_030650845.1
SO_2017
GCA_041648475.1
nzgw24
GCA_023249105.1
BLZ2
GCF_002487355.1
RU-BES
GCA_905339155.1
arrA
arxA
arrB
arrC
arrD
ubiE
glcV
tatA
coaBC
fmdE
cinA
moaA
DNA ligase
RNA helicase
truA
ribosomal genes
aroE
IS66 transposase
DNA helicase
acr
arsR
rps13p
rps4p
rps11p
pyrB
pyrI
dnaJ
dnaK
grpE
thsA
ilvC
hmgA
xpf
AbiEii toxin
srdB
srdA
MazE antitoxin
xerC recombinase
arrA/arxA
arrB/arxB
arrC/arxC
arrD/arxD
arrR/arxR
arrS/arxS
arrX/arxX
tatA
acr
contig break
2.5kb
0
Identity (%)
100
