# Supplementary material for: Arsenate reduction coupled to anaerobic oxidation of methane by members of the Methanoperedenaceae
Source: ISME J. 2026 Sep 26;20(1):wrag251. doi: 10.1093/ismejo/wrag251 (PMC13685797; doi:10.1093/ismejo/wrag251)
Supplement: Supplementary file 1 [file supplementary_material_wrag251.zip › Figure_S2.pdf]

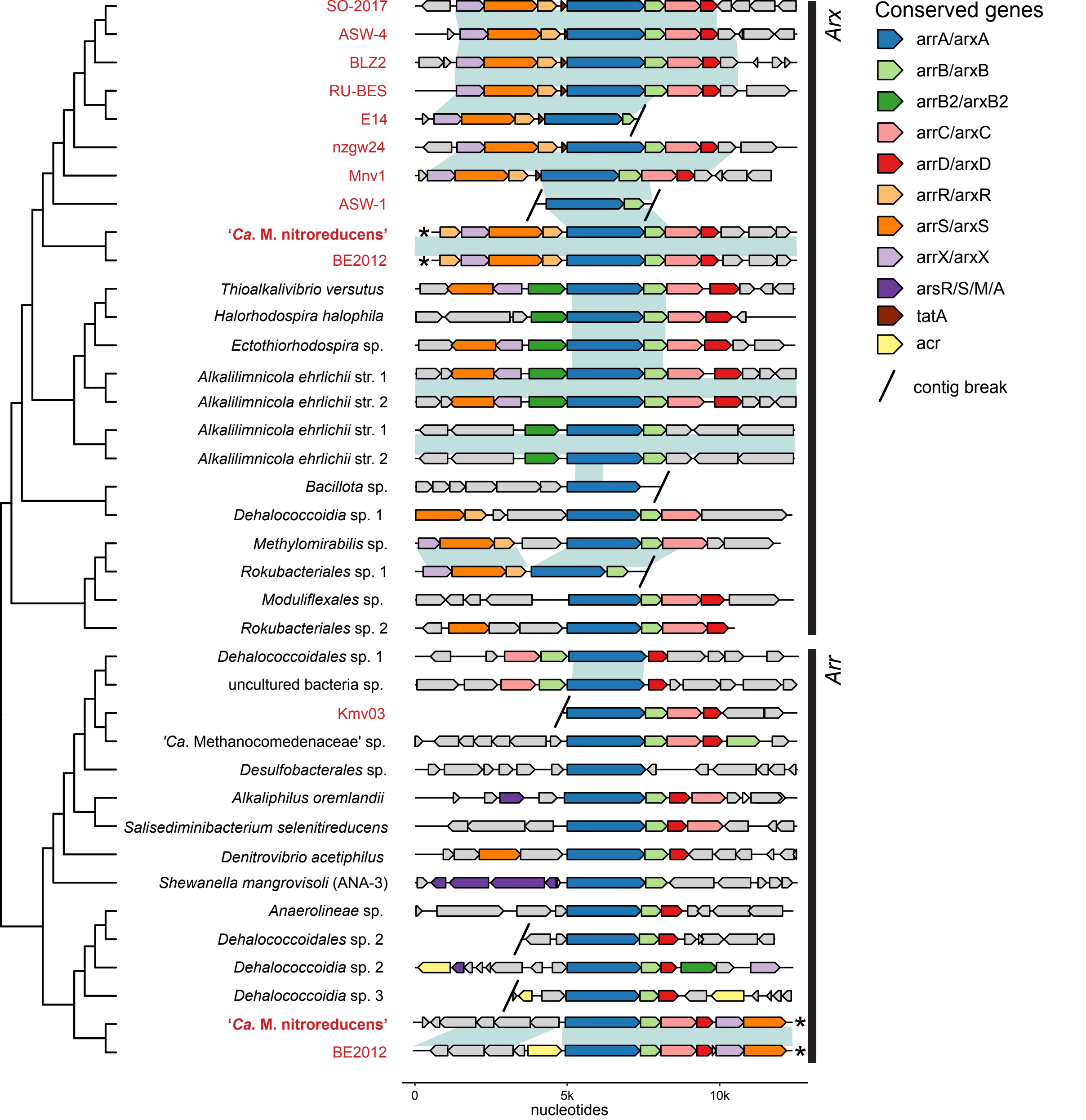

SO-2017
ASW-4
BLZ2
RU-BES
E14
nzgw24
Mnv1
ASW-1
'Ca. M. nitroreducens'
BE2012
Thioalkalivibrio versutus
Halorhodospira halophila
Ectothiorhodospira sp.
Alkalilimnicola ehrlichii str. 1
Alkalilimnicola ehrlichii str. 2
Alkalilimnicola ehrlichii str. 1
Alkalilimnicola ehrlichii str. 2
Bacillota sp.
Dehalococcoidia sp. 1
Methylomirabilis sp.
Rokubacteriales sp. 1
Moduliflexales sp.
Rokubacteriales sp. 2
Dehalococcoidales sp. 1
uncultured bacteria sp.
Kmv03
'Ca. Methanocomedenaceae' sp.
Desulfobacterales sp.
Alkaliphilus oremlandii
Salisediminibacterium selenitireducens
Denitrovibrio acetiphilus
Shewanella mangrovisoli (ANA-3)
Anaerolineae sp.
Dehalococcoidales sp. 2
Dehalococcoidia sp. 2
Dehalococcoidia sp. 3
'Ca. M. nitroreducens'
BE2012
*
Arx
Arr
0
5k
10k
nucleotides
Conserved genes
arrA/arxA
arrB/arxB
arrB2/arxB2
arrC/arxC
arrD/arxD
arrR/arxR
arrS/arxS
arrX/arxX
arsR/S/M/A
tatA
acr
contig break
