# Supplementary material for: Arsenate reduction coupled to anaerobic oxidation of methane by members of the Methanoperedenaceae
Source: ISME J. 2026 Sep 26;20(1):wrag251. doi: 10.1093/ismejo/wrag251 (PMC13685797; doi:10.1093/ismejo/wrag251)
Supplement: Supplementary file 1 [file supplementary_material_wrag251.zip › Figure_S3.pdf]

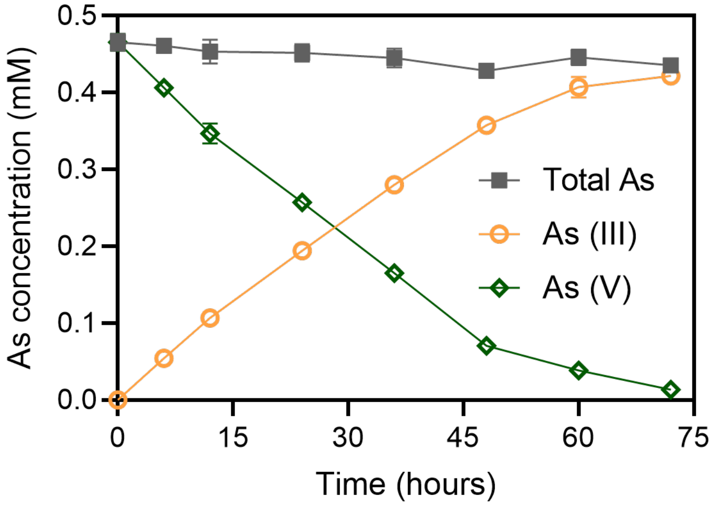

0.5
0.4
0.3
0.2
0.1
0.0
As concentration (mM)
0
15
30
45
60
75
Time (hours)
Total As
As (III)
As (V)
